# Paraoxonase 1, HDL Subclasses and Post Surgery Acute Inflammation: A Pilot Study

**DOI:** 10.3390/antiox8060192

**Published:** 2019-06-22

**Authors:** Yasmin Bains, Russell Caccavello, Kazuhiko Kotani, Alejandro Gugliucci

**Affiliations:** 1 Glycation, Oxidation and Disease Lab, Research Department, Touro California College of Osteopathic Medicine, Vallejo, CA 94592, USA; Yasmin.bains@tu.edu (Y.B.); russell.caccavello@tu.edu (R.C.); 2 Division of Community and Family Medicine, Jichi Medical University, 3311-1 Yakushiji, Shimotsuke-City, Tochigi 329-0498, Japan; kazukotani@jichi.ac.jp

**Keywords:** PON1, HDL subclasses, Acute inflammation, Chronic Inflammation, Colorectal Cancer, ApoA-1, CRP, SAA

## Abstract

High density lipoproteins (HDL) structure and function studies are needed to better understand the heterogeneous nature of the HDL particle, and its interaction with associated proteins such as apolipoprotein A-1 (ApoA-1), paraoxonase 1 (PON1) and the environment. Our study assesses the effects of acute inflammation on PON1 and HDL subclasses in post-surgical colorectal cancer patients. PON1 was measured kinetically through its arylesterase and lactonase activity and HDL sub-classes were measured using Quantimetrix Lipoprint^®^ System. White blood cells (WBC) counts, c-reactive protein (CRP) and serum amyloid A (SAA) levels were also analyzed using standard techniques. Our findings show that baseline PON1 activity is lower in colorectal cancer patients and significant reductions are observed in the acute inflammatory state post-surgery. PON1 changes are also inversely related to inflammatory markers such as SAA and CRP. In addition, our preliminary findings show that small and intermediate HDL decreases post-op Day 1. In conclusion, our study demonstrates the effects of chronic and acute inflammation on PON1. Specifically, PON1 arylesterase and lactonase activity is lower in states of chronic inflammation and further decreased in the acute inflammatory state. Additionally, in our limited sample size, while changes in PON1 and HDL subclasses may be variable in the acute inflammatory period, small HDL decreased with a loss of PON1 activity in the subacute phase.

## 1. Introduction

HDL is a heterogeneous group of particles with distinct subclasses each thought to perform specific biological functions [1]. The differing sizes, densities, lipid and protein composition, as well as their interaction with various enzymes, adds to its structural and functional diversity. Studies have found that the smaller, more dense HDL3 sub-fraction is strongly associated with cardiovascular and carotid artery disease [2,3], likely due to its more potent cholesterol efflux and antioxidant properties compared to its counterpart, HDL2 [2]. Paradoxically, despite the inverse relationship of high density lipoprotein cholesterol (HDL-C) to coronary artery disease (CAD), recent studies show that normal or increased HDL-C may not lower cardiovascular risk [4,5], and a large mean HDL size may be associated with elevated cardiovascular risk [6]. As the HDL-C hypothesis loses weight, HDL function assays are increasingly important in assessing the role of HDL in cardiovascular disease.

In addition to its reverse cholesterol transport and cholesterol efflux activity, HDL has various endothelial promoting and cardio-protective properties; it is antithrombotic, anti-inflammatory and an antioxidant [7,8,9]. PON1, an enzyme found mostly in bound form to HDL [10], in association with ApoA-1 [11], affords antioxidant capacity to HDL and may explain, in part, its anti-atherogenic function [12,13]. PON1 is a promiscuous enzyme [14]; however, its most likely physiological substrate is a lactone [10,15]. PON1 metabolizes oxidized phospholipids and prevents oxidation of low density lipoproteins (LDLs), which is cytotoxic to endothelial cells and linked to the progression of atherosclerosis [16]. ApoA-1, the major structural protein within HDL, is required to activate PON1 [17] and, therefore, proper function of both molecules is essential for HDL’s anti-oxidant and protective effects.

A thorough study of the proteins and enzymes associated with HDL subpopulations is necessary in order to better understand the diverse and complex nature of HDL’s function [18]. For example, PON1 and serum amyloid A (SAA), an acute phase protein, are inversely related and may be used as markers to assess HDL function in states of oxidative stress and inflammation [15,19]. Inflammation leads to dysfunctional HDL that is pro-atherogenic. Previous studies have assessed the effects of chronic inflammation, showing that prolonged SAA secretion by the liver is associated with lower levels of ApoA-I and PON1 activity [19,20]. However, HDL functional assays related to acute inflammation are limited. A recent meta-analysis has shown a consistent association between cancer and lower serum PON1 activities [21], so it is important to study both PON1 and HDL subclasses in this condition.

The postoperative period constitutes an acute and highly inflammatory condition. We measured PON1 activity in patients before and after colorectal surgery with a follow-up of three weeks and hypothesized that acute inflammation is inversely related to PON1 activity. We also compared PON1 activity between colon cancer patients and age and gender-matched control subjects. In addition, we hypothesized that changes in PON1 activity may be in part due to HDL remodeling and shifting of PON1 between HDL particles during periods of acute inflammation. To assess this, we performed a pilot study to analyze changes in HDL subclasses in some of the subjects during this same time period.

## 2. Materials and Methods

### 2.1. Study Subjects

All subjects and controls were recruited from Kyoto Medical Center in Kyoto, Japan. A total of 14 patients (male to female ratio 8:6, age 66 ± 12 years) who required surgery due to advanced colorectal cancer, as well as 14 age and gender-matched control subjects, were enrolled in the study. Blood was sampled at four time points: pre-surgery Day 0 and postoperative Day 1, Day 7 and Day 21. For the HDL subfractions, remaining serum from 3 controls and 5 subjects was studied at three timepoints: Day 0, 1 and 7 for 3 subjects and Day 0, 7 and 21 for 2 subjects. The controls were used for comparison in the pre-surgical phase only. Serum was frozen at −80 °C until use. All subjects gave their informed consent for inclusion before they participated in the study. The study was conducted in accordance with the Declaration of Helsinki, and the protocol was approved by the Ethics Committee of Kyoto Medical Center, IRB protocol #CL125.

### 2.2. Methods

Peripheral WBC counts, serum C-reactive protein and SAA levels were measured by standard techniques.

#### 2.2.1. PON1 Activity

##### Arylesterase

PON1 arylesterase activity was kinetically measured by following the hydrolysis of phenyl acetate at 270 nm using SpectraMax 190 Absorbance Plate Reader (Molecular Devices LLC, San Jose, USA) as follows: The reagent was prepared using 0.9 mM CaCl_2_ and 20 mM Tris with vigorous stirring at pH 8.0 w/HCL. A total of 5 µL of serum diluted in 1:5 in assay buffer was pipetted per well in triplicate to a 96-well UV flat bottom plate (Thermo 8404, Thermo Fisher, Waltham, USA). The substrate, phenyl acetate 3.4 µL, was mixed into 25 mL of arylesterase assay buffer and mixed vigorously. Following this, 200 µL of this mixture was added per well using a multichannel pipette and placed in a temperature controlled plate reader SpectraMax 190 Absorbance Plate Reader (Molecular Devices LLC, San Jose, USA) set at 25 °C. The samples were read kinetically at 270 nm for 1.5 min, every 13 s.

##### Lactonase

PON1 lactonase activity was kinetically measured using 1mM dihydrocoumarin (DHC) as a substrate at and recorded at 270 nm using a SpectraMax 190 Absorbance Plate Reader (Molecular Devices LLC, San Jose, USA) as follows: 10 µL of serum diluted 1:5 in assay buffer (50 mM Tris HCL at pH 8.0 and 1 mM CaCl_2_) and added per well in triplicate to a 96-well UV flat bottom plate (Thermo 8404, Thermo Fisher, Waltham, USA) The substrate, DHC (8.0 M), was mixed 1:1 in dimethyl sulfoxide (DMSO), vortexed and 6.25 µL of this was added to 25 mL of room temperature lactonase assay buffer and used within 5 min. A total of 200 µL of this mixture was added per well using a multichannel pipette and placed in the temperature controlled plate reader at 37 °C. Samples were read at 270 nm for 2 min 20 s, every 10 s.

Activities were reported as units per liter, where 1 U is defined as 1 mmol of substrate hydrolyzed per minute. Enzyme kinetics was calculated using SOFTmax PRO software version 5.4.6 (Molecular Devices LLC, San Jose, USA).

#### 2.2.2. HDL Subfractions

HDL subfractions were measured using the Quantimetrix Lipoprint^®^ System HDL Subfractions Kit (Quantimetrics Corporation, Redondo Beach, USA). The electrolyte buffer solution was prepared by dissolving one vial of the buffer salts in 1200 mL of distilled water. 25 µL of the sample were added to the polyacrylamide Lipoprint^®^ HDL Gel Tubes (Quantimetrics Corporation, Redondo Beach, USA). Then, 300 µL of Lipoprint^®^ HDL Loading Gel was added to each tube. A parafilm strip was placed between the gel tubes and preparation rack cover to avoid contamination. The loading gel and sample was mixed by inverting the preparation rack several times and then placed against the preparation light to allow the loading gel to photopolymerize for 35 min. Once photopolymerization was complete, the tubes were inserted into the silicone adapter so that the tubes were flush with the lower side of the adaptor. Then, 1000 mL of room temperature electrolyte buffer solution was added to the lower chamber and 200 mL to the upper chamber. A current of 3 mA per gel tube was delivered to the chambers and the voltage was set at maximum delivery (500 V) for approximately 1.5 h. The gel tubes were allowed to rest for 30 min after completion of electrophoresis. The electrophoresed HDL gels were scanned and analyzed using Lipoprint^®^ LDL/HDL subfractionation system.

### 2.3. Statistical Analysis

Data are expressed as mean ± SD. The difference in parameters was tested by the *t*-test or ANOVA for multiple points comparison. Statistical significance was set at *p* < 0.05.

## 3. Results

### 3.1. Baseline PON1 Activity is Lower in Colon Cancer Patients

**Figure 1:**
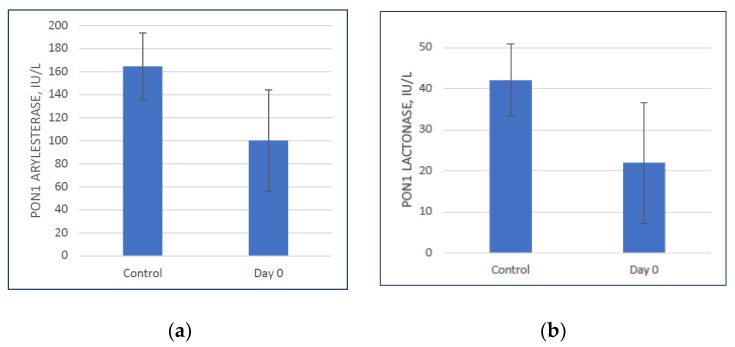
(**a**) Paraoxonase 1 (PON1) arylesterase activity (mean ± SD) in control group and experimental group Day 0 (Pre-surgery). PON1 activity is 40% lower in colon cancer patients compared to the control group (*p* = 2.19 × 10^−3^). (**b**) PON1 lactonase activity (mean ± SD) in control group and experimental group Day 0 (Pre-surgery). PON1 activity is 48% lower in colon cancer patients compared to the control group (*p* = 1.33 × 10^−3^).

The mean PON1 arylesterase and lactonase activity in our experimental group was 40% and 48% lower, respectively, compared to the control group, *p* < 0.002 (Figure 1a,b). This suggests that chronic inflammation has a negative effect on PON1 activity.

### 3.2. Significant Further Reduction in PON1 Activity Was Observed Post-Surgery

**Figure 2:**
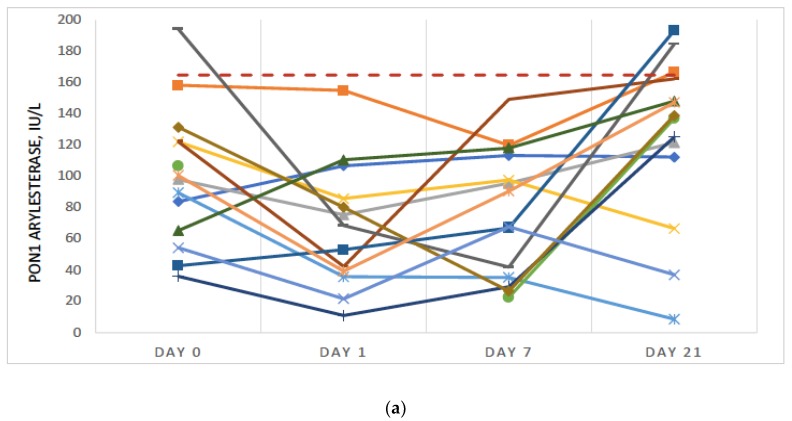

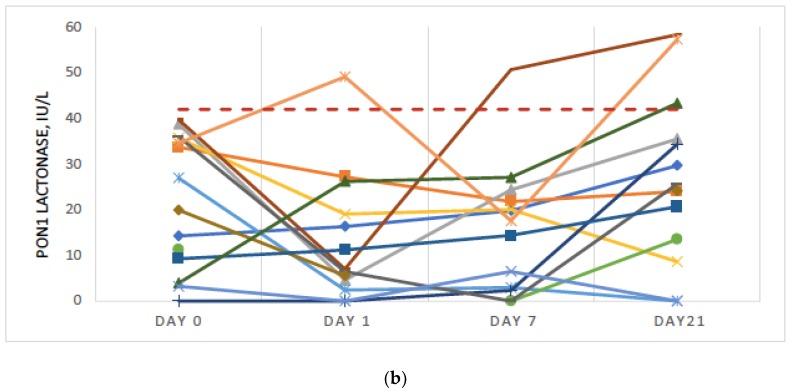
(**a**) Paraoxonase 1 (PON1) arylesterase activity (IU/L) for individual patients. For comparison, the control group average is shown as a horizontal dotted line; (**b**) PON1 Lactonase Activity (IU/L) for individual patients. For comparison, the control group average is shown as horizontal dotted line.

Ten of 14 subjects had a decrease in PON1 arylesterase activity (*p* = 2.60 × 10^−2^) and 9 of 14 had a decrease in PON1 lactonase activity (*p* = 8.57 × 10^−2^) Day 0 to Day 7 (Figure 2a,b). PON1 increased by Day 21 for most patients and in some cases, exceeded the control group averages, suggesting that improvement in PON1 may be associated with a reduction in acute inflammation. Some patients experienced a further decline in PON1 by Day 21, which may be associated with other comorbidities, late stage colorectal cancer and complications.

### 3.3. PON1 Changes Are Negatively Associated with Changes in Other Markers of Inflammation

**Figure 3:**
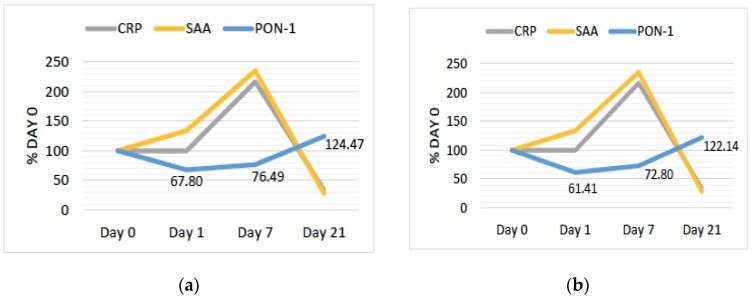
(**a**) Mean percentage change in arylesterase activity and inflammatory markers, *n* = 14; (**b**) mean percentage change in lactonase activity and inflammatory markers, *n* = 14. CRP: c-reactive protein; SAA: serum amyloid A.

As shown in Figure 3a,b, changes in PON1 arylesterase and lactonase activity are negatively associated with changes in classic inflammatory markers. We noted a sharp decline in PON1 from Day 0 to Day 1 in the subacute phase. SAA, an inflammatory marker, increased in this same time period, with a further increase from Day 1 to Day 7, during which time PON1 levels begin to increase again. CRP, another inflammatory marker, remained stable in the subacute phase but sharply increased between Day 1 and 7. Between Day 7 and Day 21—the recovery phase—SAA an CRP decreased sharply and PON1 increased to an average activity level greater than Day 0. Furthermore, mean WBC counts increased from 6.8 × 10^3^/μL to 10.2 × 10^3^/μL from Day 0 to Day 7 and decreased to 5.2 × 10^3^/μL by Day 21.

### 3.4. Small and Intermediate HDL Decrease in the Hyperacute Phase

**Figure 4:**
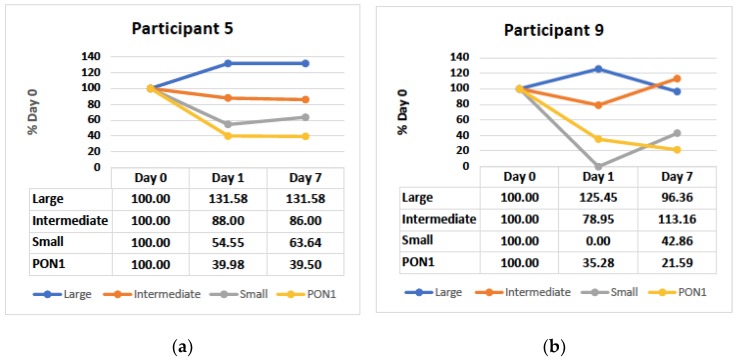
PON1 arylesterase and small, intermediate and large HDL as a percentage of Day 0 for (**a**) participant 5 (**b**) and participant 9.

During the acute inflammatory period (Day 0 to 7), changes in HDL subclasses and PON1 are variable across all 5 participants. However, in the subacute period (Day 0 to 1), we observed a decrease in both intermediate and small HDL, and an increase in large HDL for 3/3 participants (Figure 4a,b).

## 4. Discussion

This pilot study was undertaken to evaluate the effect of acute inflammation on PON1 activity and HDL subclasses. Our data report a number of significant findings: First, in our case-control study, PON1 arylesterase and lactonase activities are lower in colon cancer patients confirming the effect of cancer itself and/or of added chronic inflammation as previously shown [21]. Evaluating both arylesterase and lactonase physiological activity elucidates that the decrease in PON1 enzymatic activity is likely to be associated with enzyme structure or changes in the HDL environment, and is less likely to be associated with polymorphisms, which typically affect arylesterase but not lactonase activity. Secondly, both activities are further depressed due to acute inflammation after surgery with a recovery after 21 days. Thirdly, changes in PON1 activity parallel changes in CRP and SAA, two key sensitive markers of inflammation. Specifically, PON1 decreases with an increase in CRP and SAA. Lastly, HDL subclasses undergo changes during the acute inflammatory period, notably in the hyperacute phase, where small HDL decreases in association with a loss of PON1 activity.

Our data show that most patients had an increase in PON1 arylesterase and lactonase activity by Day 21, which suggests that antioxidant properties of HDL can potentially be restored post-colorectal surgery following an acute inflammatory period. Some subjects saw a drastic improvement by week three, where PON1 activity was higher than the control group average. Previous studies have shown that PON1 activity is lower in patients with chronic inflammation, such as in end stage renal failure [22]. In addition, the effect of various therapeutic interventions on PON1 has also been explored [20,22]. For example, hemodialysis partially restores PON1 arylesterase and lactonase activity, which may afford protection against oxidative stress and the progression of atherosclerosis in patients with end stage renal disease [22].

Studies have also shown that PON1, an antioxidant marker, and SAA, an inflammation marker, move in opposite directions and indicate dysfunctional and a more proatherogenic form of HDL [15,23]. Inflammation and oxidative stress coexist in patients with chronic disease [15,24] and under these conditions native HDL may be converted into dysfunctional HDL [7,25] with altered proteins. These changes may contribute to the less protective and more atherogenic nature of HDL [15]. Similarly, our findings in the acute inflammatory state showed that average SAA and CRP levels increased sharply from Day 1 to 7 and then decreased to below pre-surgery levels. During the same time period, average PON1 arylesterase and lactonase activity was lower compared to pre-surgery levels and increased by Day 21. Likewise, Van Lenten et al. showed that HDL lost its anti-inflammatory characteristics in the acute phase response possibly due to a loss in PON1 activity in both human and rabbit models [26]. In addition, as SAA increased during this time frame, both ApoA-1 and PON1 levels decreased [26]. The latest findings by Iftimie et al., who studied the effects of surgery on PON1 and markers of inflammation in hospitalized patients, also show lower PON1 and higher inflammatory markers (monocyte chemoattractant protein 1, CRP and procalcitonin) compared to controls, and the decrease in serum PON1 activity was maintained 14 days post-surgery [27]. Furthermore, several studies support the hypothesis that serum PON1 is decreased in states of increased inflammation and oxidative stress [28,29], and PON1 and HDL-C have shown to be inversely related to CAD [29]. Our findings, albeit in a small sample, suggest another layer of depth to these relationships, showing that though changes in HDL subclasses and PON1 may be variable during the acute inflammatory period, of note are the changes in the hyperacute phase, where small HDL decreased with a loss of PON1 activity. Further studies exploring the role of HDL subclasses and PON1 activity in the inflammatory state are needed, as this has significant implications for the prevention, pathogenesis and mortality associated with CAD [30].

Changes in HDL can also cause changes in related proteins. Studies have shown that serum ApoA-1 levels are correlated with HDL-bound PON1 arylesterase activity and PON1 associates with HDL particles that are rich in ApoA-1 or apolipoprotein E [31]. Therefore, any changes in the HDL environment can cause changes in proteins such as ApoA-1 and PON1, affecting HDL’s functional properties in the short and long term. It is already known that myeloperoxidase (MPO) promotes site-specific oxidative modification and impairs PON1 and ApoA-1 function through an HDL–MPO–PON1 ternary complex [18,32]. Further structure and function studies are critical to better understand the interaction between various proteins associated with HDL for therapeutic targets.

The limitations of our study include the relatively small sample size. While we found that changes in PON1 may be partly associated with changes in HDL environment, a larger sample size may clarify the extent to which these changes affect PON1 activity. In addition, we did not explore other factors which may be at play that cause dampened PON1 activity. These include the role of other enzymes, such as MPO, or the suppression of PON1 synthesis. The type of surgical procedure and anesthesia may also play a role in PON1 activity, as shown by Iftimie et al. [27], and patients on cholesterol lowering medications and other comorbidities such as diabetes may also have altered levels of PON1 [33]. Future studies should address these limitations to better assess how states of acute inflammation can affects PON1 in a larger population.

At this point, our findings support the contention that changes in PON1 may be only in part due to changes in HDL subclasses and that other factors such as oxidative stress or decreased PON1 synthesis may also be operating. Our preliminary data show that while HDL subclass remodeling occurs during acute inflammation, these changes do not always parallel changes in PON1 directly. An interesting finding is Day 1 post-surgery where PON1 reductions correspond to reductions in smaller HDL subclasses. Previous studies have found higher PON1 activity in smaller HDL subclasses [34,35]; therefore, there may be an association with lower PON1 and a decrease in small/intermediate HDL subclasses during acute inflammation. However, there remains some controversy as other authors have shown more PON1 activity in larger HDL particles [36,37]. Indeed, we have previously shown that PON1 activity swiftly shifts between HDL subfractions during ex vivo maturation [17]. A larger study may provide further insight.

Our data also confirm the known role of chronic inflammation on PON1 activity and extend our knowledge by showing the dramatic effects of acute inflammation on both the esterase and lactonase activities of PON1. Our results are consistent with regards to the critical role of inflammation both on HDL antioxidant function and subclasses distribution. Together with the extensive literature showing the role of inflammation in atherogenesis, our data support the contention that yet another mechanism, namely, lower PON1 protective activity, may explain dysfunctional HDL.

## 5. Conclusions

Our current data show that PON1 activity decreases further from lower baseline levels post colorectal surgery due to acute inflammation, with a recovery by Day 21. In addition, PON1 activity is negatively associated with other markers for inflammation such as CRP and SAA. Finally, small and intermediate HDL decreases with loss of PON1 activity in the hyperacute phase (Day 1). We suggest that, paired with the lipid profile, the triglyceride/HDL-C ratio, high sensitivity CRP and MPO, PON1 measurement could be a valuable addition to our diagnostic armamentarium and should be included in more clinical studies to evaluate its performance and predictive value for cardiovascular major events.
